# On the Error State Selection for Stationary SINS Alignment and Calibration Kalman Filters—Part II: Observability/Estimability Analysis

**DOI:** 10.3390/s17030439

**Published:** 2017-02-23

**Authors:** Felipe O. Silva, Elder M. Hemerly, Waldemar C. Leite Filho

**Affiliations:** 1 Department of Engineering, Federal University of Lavras, Lavras 37200-000, Brazil; 2 Aeronautics Institute of Technology, Division of Electronic Engineering, São José dos Campos 12228-900, Brazil; hemerly@ita.br; 3 National Institute for Space Research, Division of Space Mechanics and Control, São José dos Campos 12227-010, Brazil; waldclf@gmail.com

**Keywords:** SINS, alignment, calibration, error state selection, observability, estimability

## Abstract

This paper presents the second part of a study aiming at the error state selection in Kalman filters applied to the stationary self-alignment and calibration (SSAC) problem of strapdown inertial navigation systems (SINS). The observability properties of the system are systematically investigated, and the number of unobservable modes is established. Through the analytical manipulation of the full SINS error model, the unobservable modes of the system are determined, and the SSAC error states (except the velocity errors) are proven to be individually unobservable. The estimability of the system is determined through the examination of the major diagonal terms of the covariance matrix and their eigenvalues/eigenvectors. Filter order reduction based on observability analysis is shown to be inadequate, and several misconceptions regarding SSAC observability and estimability deficiencies are removed. As the main contributions of this paper, we demonstrate that, except for the position errors, all error states can be minimally estimated in the SSAC problem and, hence, should not be removed from the filter. Corroborating the conclusions of the first part of this study, a 12-state Kalman filter is found to be the optimal error state selection for SSAC purposes. Results from simulated and experimental tests support the outlined conclusions.

## 1. Introduction

Inertial navigation is the process of continuously determining the position, velocity and orientation (attitude) of a vehicle, relying solely on the information provided by rigidly-mounted (strapped down) accelerometers and angular rate sensors [1]. A strapdown inertial navigation system, or merely SINS, implements the concept presented above, by numerically integrating the raw outputs of the inertial sensors, also assuming that initial conditions for the SINS integrands are readily available [2].

For certain modern-day applications, the SINS initialization process can only occur under stationary conditions. As explained by Jekeli [3], this stationary condition allows the position and velocity initial information to be easily ascertained, but in turn, it severally hinders the determination of the vehicle initial orientation information (a process known as alignment). When the nature of the application is strategic, as for instance, in satellite launch vehicles (VLS) and ballistic missiles, it is highly desirable that the alignment is performed without any kind of external aid [4]. This “self-alignment” procedure is generally divided into two stages, the coarse and the fine alignment [5].

The coarse alignment is an analytical process that generates rough estimates of the vehicle initial orientation [6,7]. Due to some weak assumptions on which it is based, namely perfectly stationary conditions and unbiased inertial sensors, the coarse alignment generally fails to comply with the system’s accuracy requirements, and a posterior stochastic filtering and optimal estimation-based procedure becomes necessary [2]. The main purpose of this “fine alignment” stage is to improve the vehicle initial orientation information, besides estimating the uncompensated inertial sensor biases (a process known as calibration) [8].

The stationary self-alignment and calibration (SSAC) method most referred to in the literature has been established by Bar-Itzhack and Berman [9]. In their work, Bar-Itzhack and Berman employed an autonomous inertial navigator, in consonance with a linearized augmented state Kalman filter, to estimate and compensate the SINS initial attitude and the uncompensated inertial sensor biases. On the knowledge that stationary conditions applied, the “zero” velocity update (ZVU) strategy was used as the measurement input to the filter [8].

Although the SSAC strategy proposed by Bar-Itzhack and Berman had been originally derived from the full propagation error model of stationary autonomous SINS (traditionally defined with 15 states), it was a reduced-order filter (with 10 states) that was employed in their work [9]. In this filter, the position errors and the vertical channel (vertical velocity error and vertical accelerometer bias) have been purposely disregarded, under the assumption of not being of interest [9], or instead, only weakly coupled to the measurement signals [10].

When we examine how the error state selection (for SSAC purposes) has been performed in more recent works, we notice that Kalman filters with 15 states (full-order filter) [1,11,12], 12 states [13,14,15,16,17,18,19,20], 10 states (as in the Bar-Itzhack and Berman’s work) [10,21,22,23,24,25,26,27,28,29,30,31] and 9 states [32,33,34] are the most recurrent choices. Obvious questions that arise, in considering these works, are: Are these filters equivalent in terms of estimation performance? Otherwise, which is the optimal error state selection for SSAC purposes?

As suggested by Kortüm [35], the latter questions may be answered by identifying the error states whose influence on the system behavior is not significant. Additionally, an “observability/estimability analysis” of the system may provide additional insight into the problem. In the first part of this study [36], we concentrated our efforts on systematically addressing the former issue. This paper, conversely, aims to investigate the SSAC observability/estimability problem.

Since the SSAC problem consists of a linear time-invariant (LTI) estimation problem, the observability analysis does not represent a great issue by itself. As explained by Yonezawa [37], the observability evaluation of LTI systems can be directly performed through the investigation of the rank of the observability matrix, regardless of its stochastic characteristics [38]. When the SSAC problem is examined from this standpoint, an observability deficiency is found, which means that the Kalman filter state vector cannot be uniquely determined through the adopted measurements.

Despite being quite straightforward, the observability matrix rank test can only provide a “yes-no”-type answer for the complete observability of the system [32,39]. This means that it allows us to infer the number of unobservable modes (or directions) in the problem, but no clue is provided about which of these modes are, neither how “well estimated” they can be. In order to shed more light onto the problem, the so-called “degree of observability” of the system [40], whose definition is frequently associated with that of “stochastic observability” [41,42] or even with that of “estimability” [43], does also need to be investigated.

The method usually adopted to quantify the “degree of observability” of linear systems consists of monitoring the decrease in the error state covariance matrix [28,29,30,32,44,45,46,47,48,49,50,51]. As generally assumed in this “covariance analysis”, if the variance of a state diverges in time or tends to remain unchanged, the state is considered unobservable. Moreover, if an error state has its variance decreased, the faster the reduction, the higher its observability [10,28,32,47,48].

Both statements, however, when analyzed from the control theory standpoint, are found to be equally wrong, since it is entirely possible that observable error states have their variance increased, as well as unobservable states decreased [9,29]. As analyzed by Hong et al. [48], the latter is found to be particularly true if cross-correlations exist between observable and unobservable states in the initial error state covariance matrix. Moreover, the conception that a state can be “more observable” than the other is incorrect, since observability, as originally defined by Kalman [52], is a Boolean property of the system. As properly analyzed by Baram and Kailath [43], the source of the latter misconceptions derives mostly from a misinterpretation of the concepts “observability” and “estimability”, which has led authors to, not infrequently, draw misleading conclusions about filter order-reduction in practical estimation problems [32,33,34].

Another very detrimental limitation of the covariance analysis refers to the fact that it is usually limited to the mere evaluation of the terms along the major diagonal of the covariance matrix [29]. As analyzed by Ham and Brown [39], this implies the risk of overlooking some cross-correlations that may have significance for the estimation process. There are situations, for instance, where the filter is estimating linear combinations of error states quite well, but this is not apparent from a glance at the diagonal terms of the error covariance matrix.

An interesting alternative to quantify the “estimability” of linear systems has been proposed by Ham and Brown [39]. In their work, Ham and Brown demonstrated that the eigenvalues and eigenvectors of the error covariance matrix, when properly normalized, provide more insight into the estimability of linear combination of states, thereby supplying the previously commented deficiency of the covariance analysis. Ham and Brown’s estimability approach had been successfully employed for the evaluation of the multiposition alignment [53], in-flight alignment [46,54], aided alignment [55,56], alignment on a rocking base [57], INS/GNSS integration [48,58], as well as in several other applications not related to inertial navigation [59,60,61].

Concerning the SSAC problem, however, very few works in the literature have employed Ham and Brown’s approach for the sake of estimability analysis [62,63]. In the particular case of [62], Rothman et al. investigated the estimability of an inertial navigation system subject to body velocity constraints, which is slightly different from the SSAC problem (although the aforementioned authors claimed that both cases yield the same unobservable subspace). Moreover, Rothman et al. [62] have not investigated the individual estimability of the error states, but instead, uniquely of their linear combinations. Conversely, in [63], the eigenvalues and eigenvectors of the covariance matrix have only been computed for a single time instant, thereby not enabling us to properly evaluate how well estimated the error states can be during the whole SSAC process. Additionally, it is found that some conclusions of Rothman et al. [62] and Fang [63] may have been misconceived, in light of very important works traditionally established in the literature [9,14,31,48].

In this paper, hence, we systematically investigate the SSAC observability/estimability problems, still aiming at the optimal error state selection for SSAC Kalman filters. In order to characterize the unobservable modes of the problem, initially, we analytically manipulate the estimation algorithms derived in [36]. As a result of this innovative analysis, we show that the unobservable modes are, in fact, linear combinations of the alignment errors, inertial sensor biases and position errors, which consequently, are to be considered individually unobservable in the problem. Moreover, we demonstrate that the unobservable modes can be grouped into different and uncoupled subspaces.

On the basis of simulated and experimental data, covariance analyses are performed, shedding some light onto the SSAC estimability problem and allowing us to draw important conclusions about filter order reduction. The eigenvalues and eigenvectors of the normalized error state covariance matrix are also evaluated (during the whole alignment process) providing additional insight into the individual estimability of the error states.

As the main contributions of this paper, we remove some misconceptions found in the traditional literature regarding the SSAC “observability” and “estimability” deficiencies, also demonstrating that, for the sake of filter order reduction, only the latter is truly meaningful. As a straightforward consequence of the analysis, the position errors are demonstrated to be the sole “non-estimable” quantities in the SSAC problem, and hence, the sole error states liable to be neglected. We prove that, despite being unobservable, the horizontal accelerometer biases and the east angular rate sensor bias are “estimable” quantities and, consequently, should not be eliminated from the Kalman filter state vector, under the risk of impairing the overall estimation performance.

The latter verifications corroborate the conclusions achieved in the first part of this study [36], where a 12-state Kalman filter was considered the optimal error state selection for SSAC purposes. As a minor contribution of this paper, we demonstrate that the estimation accuracy of the SSAC error states is also dependent on the choice of the initial error state covariance matrix, which figures as an important subject for future investigation.

The remainder of this paper is structured as follows: Section 2 and Section 3 present an overview of the SSAC observability and estimability problems, respectively, emphasizing the significance of the latter for the sake of filter order reduction. Experimental results are provided in Section 4, whilst the conclusions and suggestions for future works are given in Section 5.

## 2. Observability Analysis

Observability, in a deterministic sense, simply means that the observation of the output over the time span (0,t) provides sufficient information to determine the initial state of the system [52,64]. According to Grewal [65], observability is a structural property of the system model, which can be expressed, at least partially, by the system dynamic and measurement matrices.

As analyzed in [36], the system dynamic matrix of the SSAC problem derives from the full propagation error dynamic model of stationary autonomous SINS, which, in state space form, can traditionally described as [36],

(1) $$\dot{x}=Ax+G_{P}n_{P}$$

with,

(2) $$x={\left[\begin{array}{ccccc} \varphi^{l} & \delta v^{l} & \delta p^{l} & \delta \omega_{bias}^{l} & \delta a_{bias}^{l} \end{array}\right]}^{T}$$

(3) $$A=\left[\begin{array}{ccccc} A_{\varphi \varphi } & A_{\varphi v} & A_{\varphi p} & -I_{3\times 3} & 0_{3\times 3}\\ A_{v\varphi } & A_{vv} & A_{vp} & 0_{3\times 3} & I_{3\times 3}\\ 0_{3\times 3} & A_{pv} & 0_{3\times 3} & 0_{3\times 3} & 0_{3\times 3}\\ 0_{3\times 3} & 0_{3\times 3} & 0_{3\times 3} & 0_{3\times 3} & 0_{3\times 3}\\ 0_{3\times 3} & 0_{3\times 3} & 0_{3\times 3} & 0_{3\times 3} & 0_{3\times 3} \end{array}\right]$$

(4) $$G_{P}=\left[\begin{array}{cc} -I_{3\times 3} & 0_{3\times 3}\\ 0_{3\times 3} & I_{3\times 3}\\ 0_{3\times 3} & 0_{3\times 3}\\ 0_{3\times 3} & 0_{3\times 3}\\ 0_{3\times 3} & 0_{3\times 3} \end{array}\right]$$

(5) $$n_{P}={\left[\begin{array}{cc} \delta \omega_{rand}^{l} & \delta a_{rand}^{l} \end{array}\right]}^{T}$$

where,

(6) $$\delta p^{l}={\left[\begin{array}{ccc} \delta L & \delta \lambda & \delta h \end{array}\right]}^{T}$$

(7) $$A_{\varphi \varphi }=\left[\begin{array}{ccc} 0 & -\Omega \sin L & 0\\ \Omega \sin L & 0 & \Omega \cos L\\ 0 & -\Omega \cos L & 0 \end{array}\right]$$

(8) $$A_{\varphi v}=\left[\begin{array}{ccc} 0 & \frac{1}{r_{\lambda s}+h} & 0\\ -\frac{1}{r_{Ls}+h} & 0 & 0\\ 0 & -\frac{\tan L}{r_{\lambda s}+h} & 0 \end{array}\right]$$

(9) $$A_{\varphi p}=\left[\begin{array}{ccc} -\Omega \sin L & 0 & 0\\ 0 & 0 & 0\\ -\Omega \cos L & 0 & 0 \end{array}\right]$$

(10) $$A_{v\varphi }=\left[\begin{array}{ccc} 0 & g_{P} & 0\\ -g_{P} & 0 & 0\\ 0 & 0 & 0 \end{array}\right]$$

(11) $$A_{vv}=\left[\begin{array}{ccc} 0 & -2\Omega \sin L & 0\\ 2\Omega \sin L & 0 & 2\Omega \cos L\\ 0 & -2\Omega \cos L & 0 \end{array}\right]$$

(12) $$A_{vp}=\left[\begin{array}{ccc} 0 & 0 & 0\\ 0 & 0 & 0\\ 0 & 0 & -\frac{2g_{P}}{R} \end{array}\right]$$

(13) $$A_{pv}=\left[\begin{array}{ccc} \frac{1}{r_{Ls}+h} & 0 & 0\\ 0 & \frac{1}{(r_{\lambda s}+h)\cos L} & 0\\ 0 & 0 & -1 \end{array}\right]$$

In (1)–(13), *x* is the Kalman filter error state vector; *A* is the Kalman filter error state dynamic matrix; $G_{P}$ is the Kalman filter process noise coupling matrix; $n_{P}$ is the Kalman filter vector of independent white process noise sources; φ is the alignment rotation error vector relating the computed navigation frame to the true navigation frame; *δ* is the designation for error quantity; ***v*** is the Earth-related vehicle velocity vector; ${\left(\right)}_{bias}$ and ${\left(\right)}_{rand}$ represent the bias and random noise components of the $\omega_{ib}$ angular rate sensor and $a_{SF}$ accelerometer measurement vectors, respectively; ${\left(\right)}^{l}$ is the designation for vectors resolved in the navigation frame; $I_{3\times 3}$ and $0_{3\times 3}$ are 3×3 identity and zero matrices, respectively; *L*, *λ*, *h* are the vehicle latitude, longitude and altitude, respectively; Ω is the magnitude of the Earth angular rate vector; $r_{Ls}$ and $r_{\lambda s}$ are the radii of curvature at the Earth surface corresponding to the vehicle latitude and longitude, respectively; $g_{P}$ is the magnitude of the plumb-bob gravity; and *R* is the magnitude of the position vector from the center of the Earth to the vehicle position.

The SSAC measurement matrix, in turn, is formed from the assumption of “zero” vehicle velocity [14,16,20,66], i.e.,

(14) $$z=Hx+G_{M}n_{M}$$

with,

(15) $$H=\left[\begin{array}{ccccc} 0_{3\times 3} & I_{3\times 3} & 0_{3\times 3} & 0_{3\times 3} & 0_{3\times 3} \end{array}\right]$$

(16) $$G_{M}=-I_{3\times 3}$$

(17) $$n_{M}=\delta v_{vib}^{l}$$

where ***z*** is the Kalman filter measurement vector; *H* is the Kalman filter measurement matrix; $G_{M}$ is the Kalman filter measurement noise coupling matrix; $n_{M}$ is the Kalman filter vector of independent white measurement noise sources; and $\delta v_{vib}$ is the vehicle “quasi-stationary” random vibration-type motion vector.

Provided *A* and *H*, the observability matrix corresponding to the SSAC problem is constructed as follows,

(18) $$O=\left[\begin{array}{c} H\\ HA\\ \vdots \\ HA^{13}\\ HA^{14} \end{array}\right]$$

According to Petch and Mintchev [38], if the rank of (18) is smaller than the order of the system (in this case, 15), the latter is not completely observable through the adopted measurements. Moreover, the difference between the order of the system and the rank of the observability matrix equals the number of linearly dependent rows/columns existing in (3) and, consequently, the number of unobservable modes (or directions) in the problem.

Substituting (3) and (15) in (18) and resorting to classical matrix rank computation techniques [67], it is possible to demonstrate that the rank of the observability matrix in the SSAC problem is nine, which means that the number of unobservable modes is six. This verification is in contrast to the work of Bar-Itzhack and Berman [9], wherein the number of unobservable modes was said to be three. This difference, however, is due to the specific error state models adopted in the works. While the full SINS error model is assumed here, a simplified 10-state error model has been preferred in [9].

Even though the number of unobservable modes can be properly determined through the observability matrix rank test, the latter tells one nothing about to which states these modes correspond. According to Rhee et al. [68], in order for a state to be observable, it should be a linear combination of observable states. Otherwise, the state is unobservable. In this sense, some insight can be provided to the problem, if we analytically manipulate the differential equations inserted into the SSAC error model. Basically, the idea is to use the measurement equations (velocity errors) and their time derivatives (admittedly observable quantities), to solve for the error states, if possible. If we succeed at proceeding as indicated, the observability properties of the SSAC error states will be readily inferred [68]. Similar approaches have been successfully employed in [26,31,33,35], restricted, however, to reduced-order SSAC dynamics (10-states).

Proceeding as suggested, the following estimation algorithms for the SSAC error states have been derived in [36] (repeated here for clarity),

(19) $$\varphi_{N}=-\frac{1}{g_{P}}\left(\delta {\dot{v}}_{E}-2\Omega \sin L\delta v_{N}-2\Omega \cos L\delta v_{D}-\delta a_{biasE}\right)$$

(20) $$\varphi_{E}=\frac{1}{g_{P}}\left(\delta {\dot{v}}_{N}+2\Omega \sin L\delta v_{E}-\delta a_{biasN}\right)$$

(21) $$\varphi_{D}=\frac{1}{\Omega \cos L}\left[\frac{1}{g_{P}}\delta {\ddot{v}}_{N}+\frac{3\Omega \sin L}{g_{P}}\delta {\dot{v}}_{E}+\left(\frac{1}{r_{Ls}+h}-\frac{2\Omega^{2}\sin^{2}L}{g_{P}}\right)\delta v_{N}-\frac{\Omega^{2}\sin2L}{g_{P}}\delta v_{D}+\delta \omega_{biasE}-\frac{\Omega \sin L}{g_{P}}\delta a_{biasE}\right]$$

(22) $$\delta \omega_{biasN}=\frac{1}{g_{P}}\delta {\ddot{v}}_{E}-\frac{3\Omega \sin L}{g_{P}}\delta {\dot{v}}_{N}-\frac{2\Omega \cos L}{g_{P}}\delta {\dot{v}}_{D}+\left(\frac{1}{r_{\lambda s}+h}-\frac{2\Omega^{2}\sin^{2}L}{g_{P}}\right)\delta v_{E}-\Omega \sin L\delta L+\frac{\Omega \sin L}{g_{P}}\delta a_{biasN}$$

(23) $$\delta \omega_{biasE}=-\frac{1}{g_{P}}\delta {\ddot{v}}_{N}-\frac{3\Omega \sin L}{g_{P}}\delta {\dot{v}}_{E}+\Omega \cos L\varphi_{D}+\left(\frac{2\Omega^{2}\sin^{2}L}{g_{P}}-\frac{1}{r_{Ls}+h}\right)\delta v_{N}+\frac{\Omega^{2}\sin2L}{g_{P}}\delta v_{D}+\frac{\Omega \sin L}{g_{P}}\delta a_{biasE}$$

(24) $$\begin{array}{c} \delta \omega_{biasD}=-\frac{1}{g_{P}\Omega \cos L}\delta {\dddot{v}}_{N}-\frac{3\tan L}{g_{P}}\delta {\ddot{v}}_{E}\;\\ +\left(\frac{2\Omega \sin^{2}L}{g_{P}\cos L}-\frac{1}{\Omega \cos L(r_{Ls}+h)}-\frac{\Omega \cos L}{g_{P}}\right)\delta {\dot{v}}_{N}+\frac{2\Omega \sin L}{g_{P}}\delta {\dot{v}}_{D}\;\\ -\left(\frac{\Omega^{2}\sin2L}{g_{P}}+\frac{\tan L}{r_{\lambda s}+h}\right)\delta v_{E}-\Omega \cos L\delta L+\frac{\Omega \cos L}{g_{P}}\delta a_{biasN} \end{array}$$

(25) $$\delta a_{biasN}=\delta {\dot{v}}_{N}-g_{P}\varphi_{E}+2\Omega \sin L\delta v_{E}$$

(26) $$\delta a_{biasE}=\delta {\dot{v}}_{E}+g_{P}\varphi_{N}-2\Omega \sin L\delta v_{N}-2\Omega \cos L\delta v_{D}$$

(27) $$\delta a_{biasD}=\delta {\dot{v}}_{D}+2\Omega \cos L\delta v_{E}+\frac{2g_{P}}{R}\delta h$$

(28) $$\begin{array}{c} \delta L=\frac{1}{\Omega \sin L}[\frac{1}{g_{P}}\delta {\ddot{v}}_{E}-\frac{3\Omega \sin L}{g_{P}}\delta {\dot{v}}_{N}-\frac{2\Omega \cos L}{g_{P}}\delta {\dot{v}}_{D}\;\\ +\left(\frac{1}{r_{\lambda s}+h}-\frac{2\Omega^{2}\sin^{2}L}{g_{P}}\right)\delta v_{E}-\delta \omega_{biasN}+\frac{\Omega \sin L}{g_{P}}\delta a_{biasN}] \end{array}$$

(29) $$\begin{array}{c} \delta \lambda =-\frac{1}{\Omega \cos L}[\frac{1}{g_{P}\Omega \cos L}\delta {\dddot{v}}_{N}+\frac{3\tan L}{g_{P}}\delta {\ddot{v}}_{E}\;\\ +\left(\frac{\Omega \cos L}{g_{P}}+\frac{1}{\Omega \cos L(r_{Ls}+h)}-\frac{2\Omega \sin^{2}L}{g_{P}\cos L}\right)\delta {\dot{v}}_{N}-\frac{2\Omega \sin L}{g_{P}}\delta {\dot{v}}_{D}\;\\ +\left(\frac{\Omega^{2}\sin2L}{g_{P}}-\frac{\tan L}{r_{\lambda s}+h}\right)\delta v_{E}+\delta \omega_{biasD}-\frac{\Omega \cos L}{g_{P}}\delta a_{biasN}] \end{array}$$

(30) $$\delta h=-\frac{R}{2g_{P}}\left(\delta {\dot{v}}_{D}+2\Omega \cos L\delta v_{E}-\delta a_{biasD}\right)$$

where the subscripts *N*, *E* and *D* indicate vector components in the north, east and down directions, respectively.

As (19)–(30) indicate, besides the velocity errors and their time derivatives, all SSAC error states are linear combinations of variables whose observability cannot be guaranteed at this time. In the particular case of (19), for instance, $\varphi_{N}$ is found to be coupled to $\delta a_{biasE}$, whose observability condition is uncertain. In an attempt to solve the latter problem, let us temporarily replace $\varphi_{N}$ by $\varphi_{N}^{*}$ in (26), i.e.,

(31) $$\delta a_{biasE}=\delta {\dot{v}}_{E}+g_{P}\varphi_{N}^{*}-2\Omega \sin L\delta v_{N}-2\Omega \cos L\delta v_{D}$$

Substituting now (31) in (19) yields,

(32) $$\varphi_{N}=-\frac{1}{g_{P}}\left(\delta {\dot{v}}_{E}-2\Omega \sin L\delta v_{N}-2\Omega \cos L\delta v_{D}-\delta {\dot{v}}_{E}-g_{P}\varphi_{N}^{*}+2\Omega \sin L\delta v_{N}+2\Omega \cos L\delta v_{D}\right)$$

After the cancellation of like terms,

(33) $$\varphi_{N}=\varphi_{N}^{*}$$

Equation (33) is the analytical proof that (19) and (26) are linearly dependent expressions, which implies that $\varphi_{N}$ and $\delta a_{biasE}$ cannot be uniquely determined by the filter (by definition, hence, $\varphi_{N}$ and $\delta a_{biasE}$ are individually unobservable error states). This deficiency had already been pointed out in the work of Jiang and Lin [10]. If we carefully analyze the remaining estimation algorithms given in (19) to (30), we will notice that the same pattern is repeated for (20) and (25); (21) and (23); (22) and (28); (24) and (29); (27) and (30), respectively. The conclusion that follows is: except for the velocity errors, all SSAC error states are individually unobservable and can be grouped into the following subspaces,

(34) $$s_{1}=\left\{\begin{array}{cc} \varphi_{N}, & \frac{1}{g_{P}}\delta a_{biasE} \end{array}\right\}$$

(35) $$s_{2}=\left\{\begin{array}{cc} \varphi_{E}, & -\frac{1}{g_{P}}\delta a_{biasN} \end{array}\right\}$$

(36) $$s_{3}=\left\{\begin{array}{cc} \varphi_{D}, & \frac{1}{\Omega \cos L}\delta \omega_{biasE} \end{array}\right\}$$

(37) $$s_{4}=\left\{\begin{array}{ccc} \delta L, & -\frac{1}{\Omega \sin L}\delta \omega_{biasN}, & \frac{1}{\Omega \cos L}\delta \omega_{biasD} \end{array}\right\}$$

(38) $$s_{5}=\left\{\begin{array}{cc} \delta h, & \frac{R}{2g_{P}}\delta a_{biasD} \end{array}\right\}$$

Equations (34)–(38) constitute what we call here “subspaces of individually unobservable error states”, or merely “unobservable subspaces” (for the sake of simplicity). These subspaces, which had not been reported yet in the literature, complement the work of Jiang and Lin [10,31], wherein the number of uncoupled SSAC unobservable subspaces was said to be two.

Looking at a glance at (34)–(38), however, we could be led to infer that the obtained subspaces are not in agreement with the observability matrix rank test conducted at the beginning of this section, where the number of unobservable modes in the SSAC problem was found to be six. Let us recall, however, that we have not considered yet the longitude error, which is an error state completely decoupled from the SINS error model and for which no estimation algorithm is expected to exist (see [36] for details). Hence, it is intuitive to infer that the last unobservable mode in the SSAC problem is actually the own longitude error, i.e.,

(39) $$s_{6}=\left\{\begin{array}{c} \delta \lambda \end{array}\right\}$$

As can be noticed, the unobservable modes of the SSAC problem are not linear combinations uniquely of the alignment errors and the inertial sensor biases, as claimed in [10,14], but also of the position errors, which figures as another novelty of the presented analysis. Moreover, it is found that the formed subspaces are completely uncoupled, which means that only error states belonging to the same subspace are expected to interfere on the observability of the other.

At this point of the analysis, an important consideration is required: despite the impossibility of jointly determining the states belonging to Subspaces (34)–(39), it does not mean that this will always be true for the SSAC problem. According to Bar-Itzhack and Berman [9], the results obtained so far only allow us to say that this will happen when the chosen measurements are the vehicle velocity errors. Besides the latter, there are several possible sets of measurements, which, if performed, could yield a completely observable system [23,35,55,56,69]. The investigation of such possible sets, however, is not the purpose of this paper.

Although the observability analysis based on the analytical manipulation of the SINS error model has shed some light onto the problem, the structural considerations made so far are still somewhat abstract since they did not allow us to solve the main issue we proposed to investigate: the optimal error state selection for SSAC purposes.

As traditionally established in the literature, estimation performance stands for complete observability, which means that the existence of unobservable modes in an estimation problem is, at least, undesirable. In order to achieve complete observability, the authors have, not infrequently, “shrunk” their filters, by dropping states that they considered unobservable [32,33,34]. When the concerned problem has clearly defined unobservable states, the latter procedure is straightforward, but, when the unobservable modes are linear combinations of states (as is the case of the SSAC problem), how does one perform the filter order-reduction?

As suggested by Grewal et al. [65], combining states in order to produce a near-optimal estimation scheme is an option, which however, is limited to cases where the states are not individually required to perform posterior “correction reset” operations (of the navigation parameters, for instance). As an alternative option, some authors have tried to classify which states are “less identifiable” into their respective unobservable subspaces, attributing to them the unobservability deficiency of the system. In this sense, different authors have identified different states as unobservable [9,10,28].

**Figure 1:**
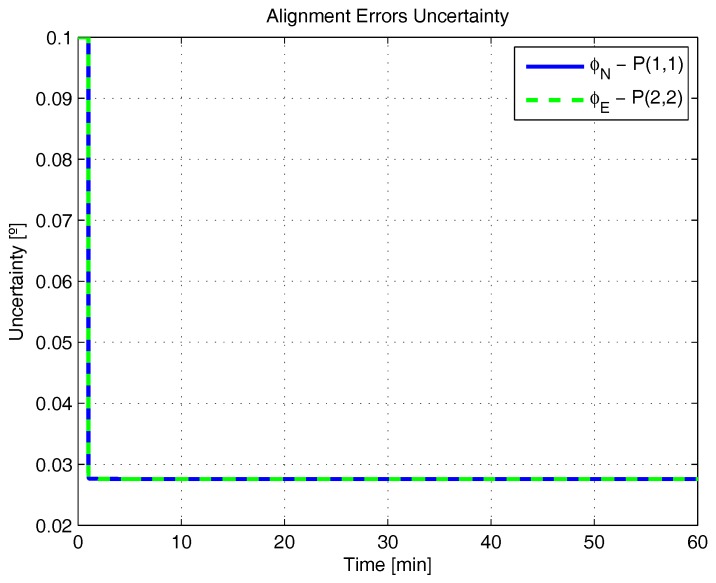
Alignment errors uncertainty in the simulated test.

As properly analyzed by Farrel and Barth [5], however, the designer does not have the freedom to “choose” which states are observable in an estimation problem. Moreover, if the designer drops either variable in an attempt to estimate the other, the reduced-order estimation problem may appear to become observable, but the resulting estimate of the retained variable will be biased from the true value of the neglected variable. For the particular case of the SSAC problem, for instance, Figures 1–3 of [36] clearly corroborate Farrel and Barth’s statement.

As it is straightforward to conclude, hence, “observability” itself may not be a strong and sufficient condition to endorse filter order-reduction in practical estimation problems. Instead, the concept of “estimability”, to be presented in sequence, seems to be a much more adequate and robust property for such purposes.

## 3. Estimability Analysis and Simulation Results

As originally defined by Baram and Kailath [43], the concept of “estimability” may be regarded as an extension to the stochastic case of the concept of observability, which, in the deterministic sense, means that the state can be completely deduced from past observations and inputs. Still according to Baram and Kailath, we shall say that a stochastic linear system is estimable if, in estimating its state from its output, the posterior error covariance matrix is strictly smaller than the prior state covariance matrix. Due to the proximity of definitions, estimability has been often associated with the concept of “stochastic observability” [41,42], as well as with that of “degree of observability”, originally proposed by Brown [40]. The most important practical consequence of Baram and Kailath’s work is: the notion of estimability is independent of that of observability, in the sense that one does not imply the other. Works that support this are [43,46,49,70]. Moreover, it is found that the adequacy of reducing the filter dimensionality in state estimation problems is much more related to the concept of estimability than to that of observability. According to Baram and Kailath [43], it is when the system is not estimable that the corresponding Kalman filter can be reduced to a lower order estimator of the state process.

As introduced in Section 1, the most employed tool for evaluating the estimability of linear systems is the “covariance analysis” [28,29,30,32,44,45,46,47,48,49,50,51]. With respect to the latter, it is usual to admit that, if all error states are jointly Gaussian and initially uncorrelated with one another, their variances can be directly inferred from the terms along the major diagonal of the covariance matrix. A major handicap of the covariance analysis, however, is that it lacks a solid analytical basis [14], which has compelled the authors to seek numerical computation support for evaluating the latter [39,48,54,55,56,57,58,61,62,63]. Similarly, in order to ascertain the estimability of the SSAC problem, let us initially consider a simulated test.

The simulated test consisted of implementing the full-order SSAC Kalman filter (15-states) described in [71], at a cadence of 1 Hz, during a period of 60 min. The simulated SINS was supposed to be located in a place with −23${}^{\circ }$12${}^{\prime }$47${}^{\prime \prime }$ of latitude, −45${}^{\circ }$51${}^{\prime }$38${}^{\prime \prime }$ of longitude and 629 m of altitude. The angular rate sensor and accelerometer datasets were generated at a cadence of 100 Hz and intentionally corrupted with 0.5${}^{\circ }$/h and 0.5 mg of constant biases and 0.0002${}^{\circ }$/$\sqrt{h}$ and with 0.01 mg/$\sqrt{Hz}$ of random walks, respectively. Moreover, the simulated SINS was considered to be perfectly stationary and aligned to the navigation frame. For the purpose of the test, the following values were assigned to the ${\hat{x}}_{0}$ initial error state vector, $P_{0}$ initial error state covariance matrix, $Q_{P}$ process noise density matrix and the $R_{M}$ measurement noise covariance matrix of the Kalman filter,

(40) $${\hat{x}}_{0}=0_{15\times 1}$$

(41) $$\begin{array}{c} P_{0}=\mathrm{diag}[{\left(0.1^{\circ }\right)}^{2}\;{\left(0.1^{\circ }\right)}^{2}\;{\left(5^{\circ }\right)}^{2}\;{\left(0.1\;m/s\right)}^{2}\;{\left(0.1\;m/s\right)}^{2}\;\\ {\left(0.1\;m/s\right)}^{2}\;{\left(0.0001^{\circ }\right)}^{2}\;{\left(0.0001^{\circ }\right)}^{2}\;{\left(10\;m\right)}^{2}\;{\left(0.5^{\circ }/h\right)}^{2}\;\\ {\left(0.5^{\circ }/h\right)}^{2}\;{\left(0.5^{\circ }/h\right)}^{2}\;{\left(0.5\;\mathrm{mg}\right)}^{2}\;{\left(0.5\;\mathrm{mg}\right)}^{2}\;{\left(0.5\;\mathrm{mg}\right)}^{2}] \end{array}$$

(42) $$\begin{array}{c} Q_{P}=\mathrm{diag}[{\left(0.0002^{\circ }/\sqrt{h}\right)}^{2}\;{\left(0.0002^{\circ }/\sqrt{h}\right)}^{2}\;{\left(0.0002^{\circ }/\sqrt{h}\right)}^{2}\;\\ {\left(0.01\;\mathrm{mg}/\sqrt{\mathrm{Hz}}\right)}^{2}\;{\left(0.01\;\mathrm{mg}/\sqrt{\mathrm{Hz}}\right)}^{2}\;{\left(0.01\;\mathrm{mg}/\sqrt{\mathrm{Hz}}\right)}^{2}] \end{array}$$

(43) $$R_{M}=0_{3\times 3}$$

**Figure 2:**
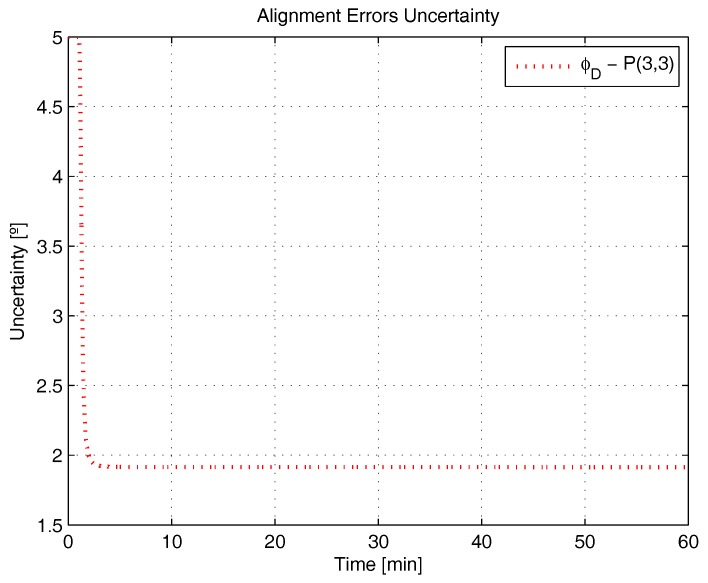
Alignment errors uncertainty in the simulated test, continuance.

**Figure 3:**
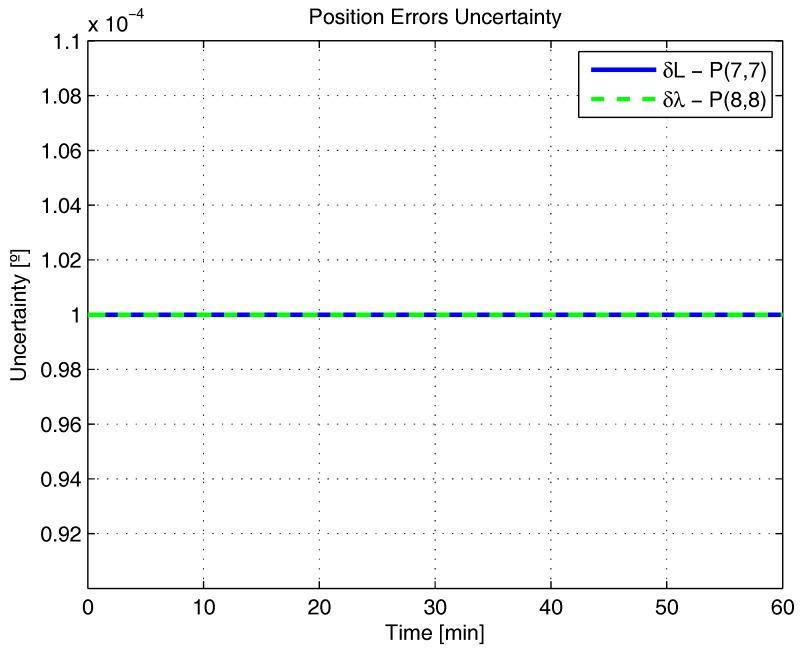
Position errors uncertainty in the simulated test.

**Figure 4:**
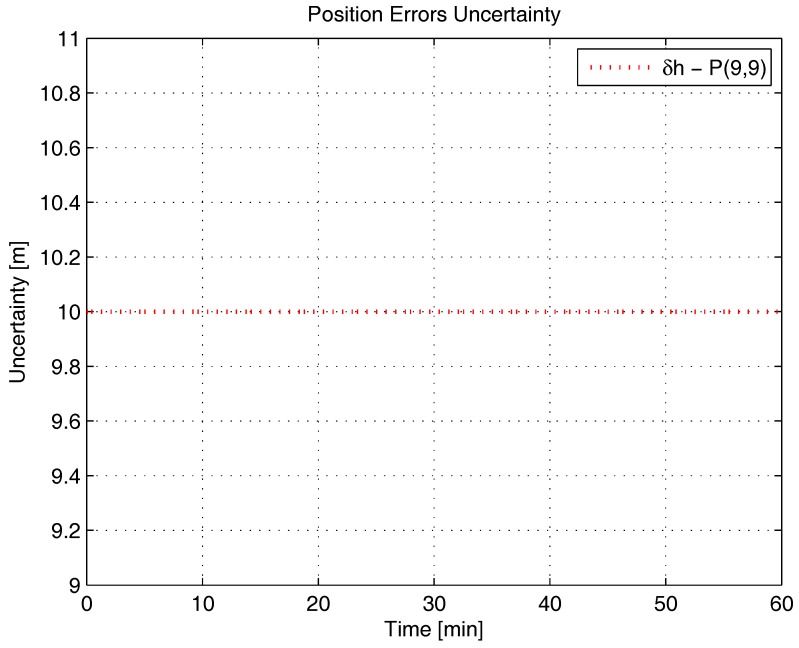
Position errors uncertainty in the simulated test, continuance.

**Figure 5:**
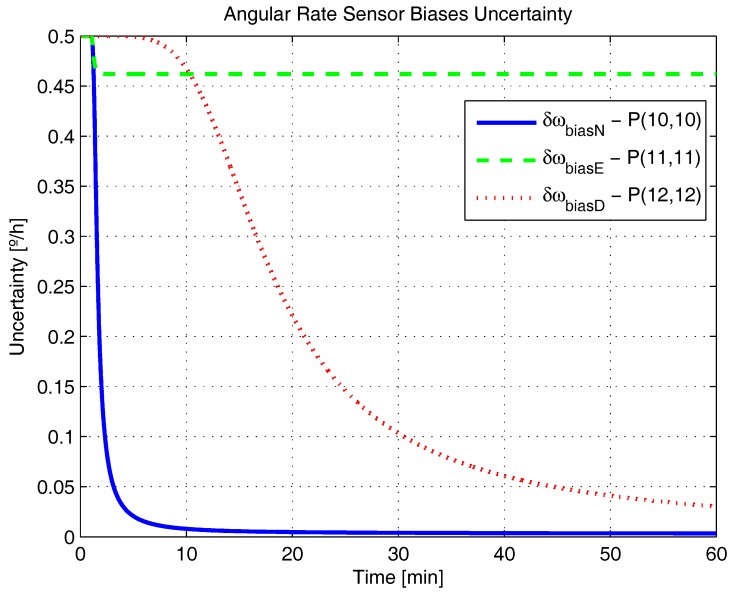
Angular rate sensor biases uncertainty in the simulated test.

**Figure 6:**
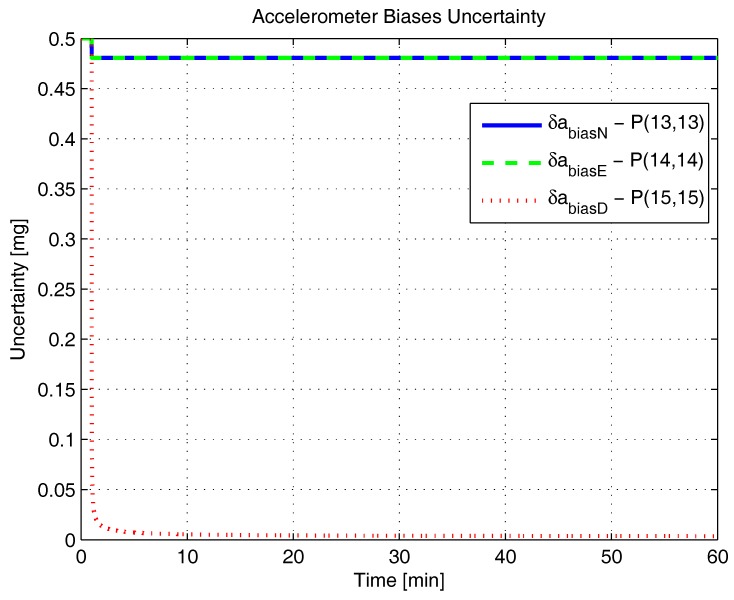
Accelerometer biases uncertainty in the simulated test.

Figure 1, Figure 2, Figure 3, Figure 4, Figure 5 and Figure 6 represent the uncertainties in the SSAC error states, as derived from the terms along the major diagonal of the covariance matrix.

As can be seen, all alignment and inertial sensor errors had their variances decreased in time (even than slightly), which means, from the estimability standpoint, that all of these states are “estimable” in the SSAC problem. This verification is in disagreement with traditional works in the area [10,24,26,27,28,31,32,33,72], wherein the horizontal accelerometer biases and the east angular rate sensor bias were said to be “non-estimable” in the problem (mainly because these authors analyzed the problem exclusively from the observability perspective).

As Figure 3 and Figure 4 clearly indicate, the sole error states admittedly “non-estimable” in the SSAC problem are the position errors, which were experimented with no decrease in their variances. When examined from the analytical standpoint, this verification does not represent a novelty, since it is already known that position errors cannot be determined uniquely through the observation of velocity measurements (recall that position errors are computed through the integration of velocity errors plus an integration constant, which the filter is unable to ascertain). Works that support this verification are [11,38,62,73].

By recalling Baram and Kailath’s statement that reduced-order filters are to be implemented based on the non-complete estimability of the system (and not on its non-complete observability), it is straightforward to conclude that the position errors are the sole state variables liable to be dropped from the SSAC Kalman filter (by inspection, they are the sole error states whose elimination is necessary to yield a completely estimable system). In this sense, when authors, as Cho et al. [32,34], Xinlong [33], Chang et al. [21], and many others [9,10,22,23,24,25,26,27,28,29,30,31], removed from their filters states other than the position errors, they seriously impaired their estimation performance. According to Savage [74], an important rule to bear in mind during the design process of Kalman filters is that error states to be included are those that significantly affect the measurement (i.e., error states that are “estimable”). Not including such states can result in misestimating other error states that have been included in the filter’s model, due to the “filter misinterpretation” of measurement data input signatures. In fact, we have observed this behavior in [36], when further reduced Kalman filters (with nine states and 10 states) were proven to be inadequate for SSAC purposes (in detriment to the 12-state filter, which stands, hence, as the optimal error state selection for SSAC purposes).

Despite the relevance of the just conducted analysis, no clue has been provided yet concerning “how rapidly” and “how accurately” determined the “estimable” error states (alignment errors and inertial sensor biases) can be in the SSAC problem. As is usual to assume in the covariance analysis [10,28,29,32,43,47,48], the latter characteristics are directly related to the convergence rate and steady state values of the diagonal terms of the error state covariance matrix.

Concerning the convergence rate of the error state variances, it is possible to notice from Figure 1 and Figure 6 that the uncertainties of $\varphi_{N}$, $\varphi_{E}$, $\delta a_{biasN}$, $\delta a_{biasE}$ and $\delta a_{biasD}$ decreased very rapidly in the first instants of the simulation, exactly as predicted in [36] (based on the estimation algorithms derived for these variables). The variances of $\varphi_{D}$, $\delta \omega_{biasN}$ and $\delta \omega_{biasE}$, in turn, did not decrease as rapidly, reaching their steady state values only after about 10 min. Concerning the variance of $\delta \omega_{biasD}$ finally, we notice that it reduced still more slowly, in agreement with the necessity of taking higher velocity error derivatives (see (24) for details). As indicated in Figure 5, this error state is expected to be recognizable only after 30 to 40 min of the simulation.

With respect to the steady state values achieved by the error state variances, the first remark we can make is that none of them reach zero. As explained by Kortüm [35], this is due to the fact that we are dealing with a non-deterministic system, i.e., a system containing noise sources, which is consistent with practical applications. According to Goshen-Meskin and Bar-Itzhack [20,75], the lower limit reached by the error state variances may be set both by the noise level experienced by the system and the existence of the unobservable modes in the latter. At a glance, it is not straightforward to decide about the influence of each of these constraints in the problem. In the work of Jekeli [3] for instance, it is stated that the limiting standard deviations of $\varphi_{D}$ and $\delta \omega_{biasE}$ depend essentially on the minimum of their respective initial standard deviations. For the particular case of the simulated test discussed here, Jekeli’s statement is translated, in mathematical notation, as follows,

(44) $$\sigma_{\varphi_{D}}\left(t_{max}\right)=\frac{\sigma_{\delta \omega_{biasE}}\left(t_{max}\right)}{\Omega \cos L}\approx \min{\left(\sigma_{\varphi_{D}},\frac{\sigma_{\delta \omega_{biasE}}}{\Omega \cos L}\right)}_{t=0}\approx 2.0724^{\circ }$$

which is, by inspection, consistent with (36).

When we refer to Figure 2, we notice that the final uncertainty of the down alignment error (P(3,3) diagonal term of the covariance matrix) nicely reproduces the steady state value predicted for it in (44). However, when we refer to the real accuracy achieved by this state in the simulated test (see Figure 3 of the first part of this study [36]), we notice that it does not match the predicted value, which is clear evidence of the covariance analysis deficiency commented on in Section 1 (namely, that diagonal terms of the covariance matrix do not rigorously correspond to the estimation accuracy of individually unobservable states or, in other words, to the accuracy of linear combination of states).

An interesting alternative to the estimability analysis of LTI systems, as also introduced in Section 1, consists of numerically computing the eigenvalues and eigenvectors of the properly normalized error state covariance matrix. This approach, originally proposed by Ham and Brown [39], provides more insight into the estimability of linear combination of states, being thus suitable for the SSAC problem. According to Ham and Brown [39], the largest eigenvalue of the covariance matrix corresponds to the (actual) variance of the state (or linear combination of states) that is poorly estimable relative to the direction of poor estimability (which is given by the corresponding eigenvector). Conversely, the state (or linear combination of states) that is most estimable is indicated by the smallest eigenvalue/eigenvector of the covariance matrix [39].

As analyzed by Ham and Brown [39], however, if the covariance matrix eigenvalues are directly calculated, without any sort of normalization, the resulting range of values is almost anything, which can cause confusion when making comparisons among the eigenvalues. In order to avoid the latter, Ham and Brown proposed a normalization scheme whereby the resulting values are calculated relative to the initial conditions of the system. Besides forcing the estimation error vector to be dimensionless, the proposed scheme sets a bound on the covariance matrix eigenvalues, constraining the latter to range between zero and the order of the system. In equations,

(45) $$P^{N+}\left(t\right)=\frac{o}{\mathrm{Tr}\left[P^{{}^{\prime }+}\left(t\right)\right]}P^{{}^{\prime }+}\left(t\right)$$

with,

(46) $$P^{{}^{\prime }+}\left(t\right)={\left(\sqrt{P_{0}^{-}}\right)}^{-1}P^{+}\left(t\right){\left(\sqrt{P_{0}^{-}}\right)}^{-1}$$

where *P* is the Kalman filter error state covariance matrix; $P_{0}$ is its initial value; superscripts ${\left(\right)}^{N}$, ${\left(\right)}^{\prime }$ and ${\left(\right)}^{+}$ designate normalized matrix, semi-normalized matrix and matrix value immediately after (“a posteriori”) the application of Kalman filter resets, respectively; *o* corresponds to the order of the system; Tr designates the trace of a matrix; and ()(t) represents a parameter calculated at time *t*.

**Figure 7:**
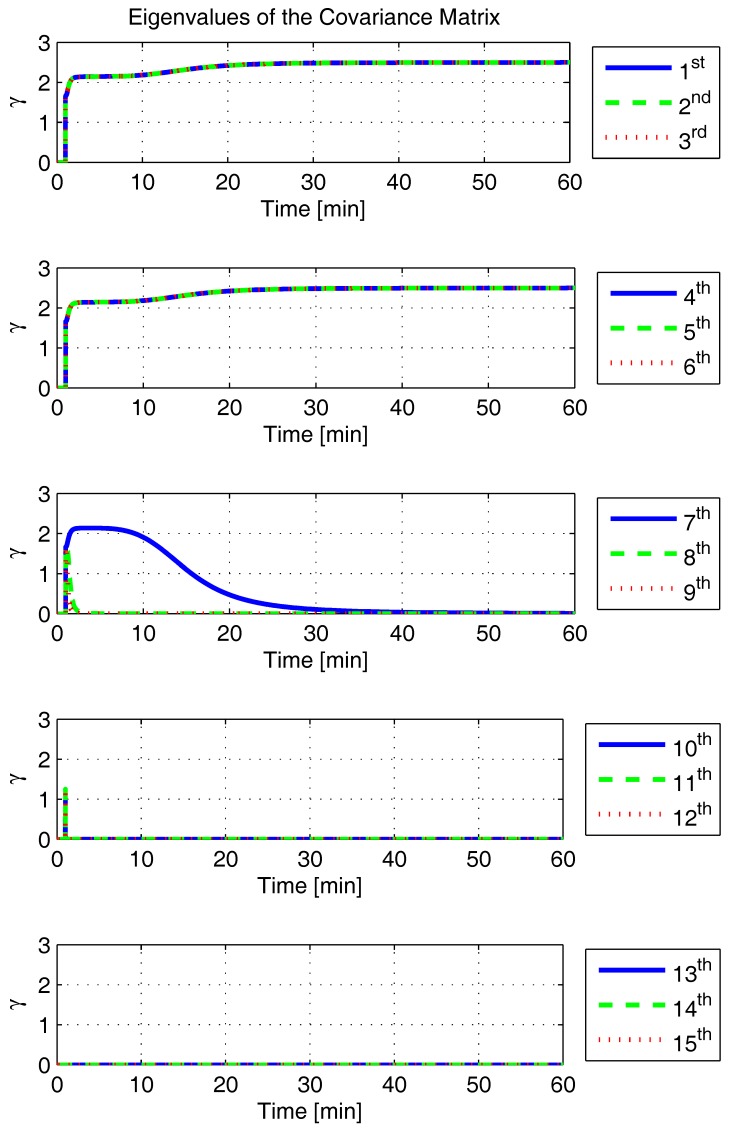
Eigenvalues of the covariance matrix in simulated test.

**Figure 8:**
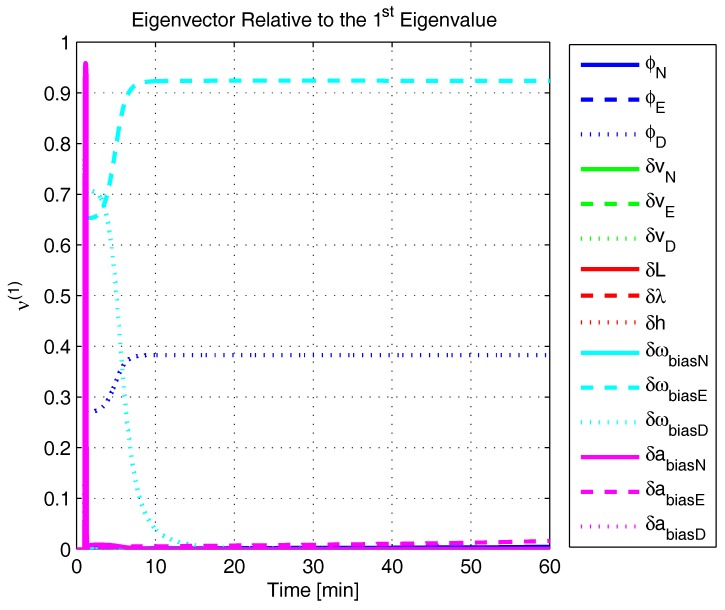
Eigenvector relative to the first eigenvalue in the simulated test.

**Figure 9:**
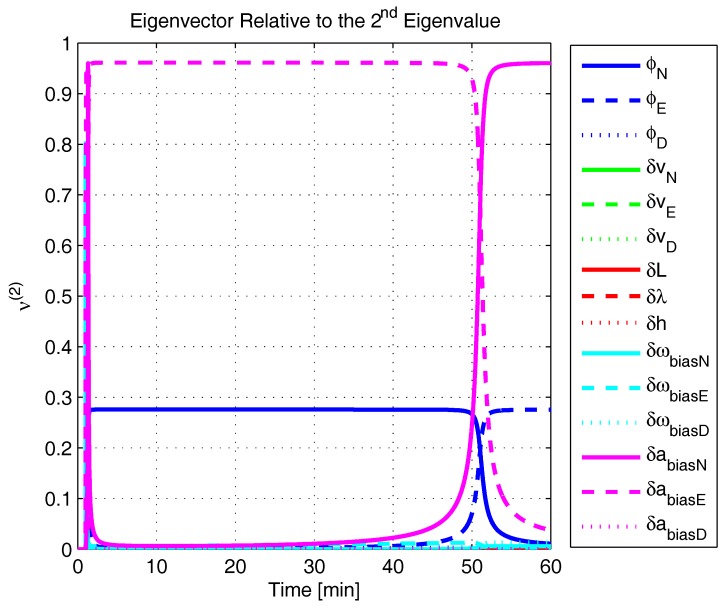
Eigenvector relative to the second eigenvalue in the simulated test.

**Figure 10:**
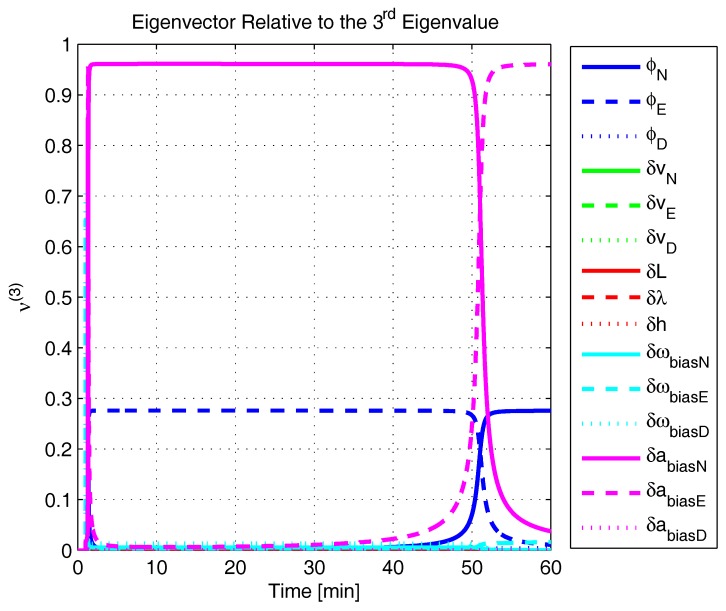
Eigenvector relative to the third eigenvalue in the simulated test.

**Figure 11:**
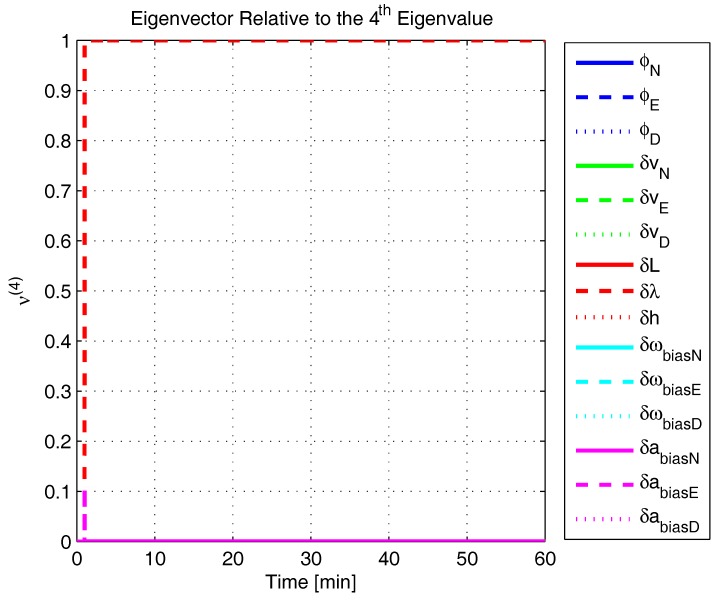
Eigenvector relative to the fourth eigenvalue in the simulated test.

**Figure 12:**
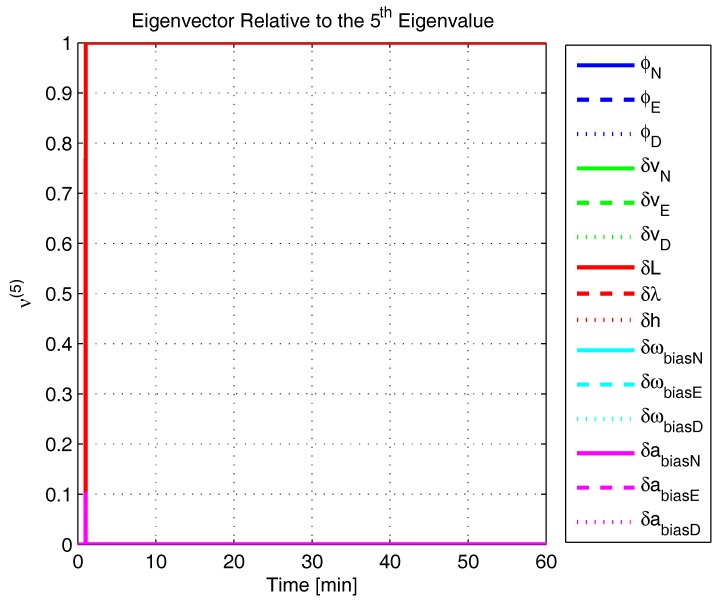
Eigenvector relative to the fifth eigenvalue in the simulated test.

**Figure 13:**
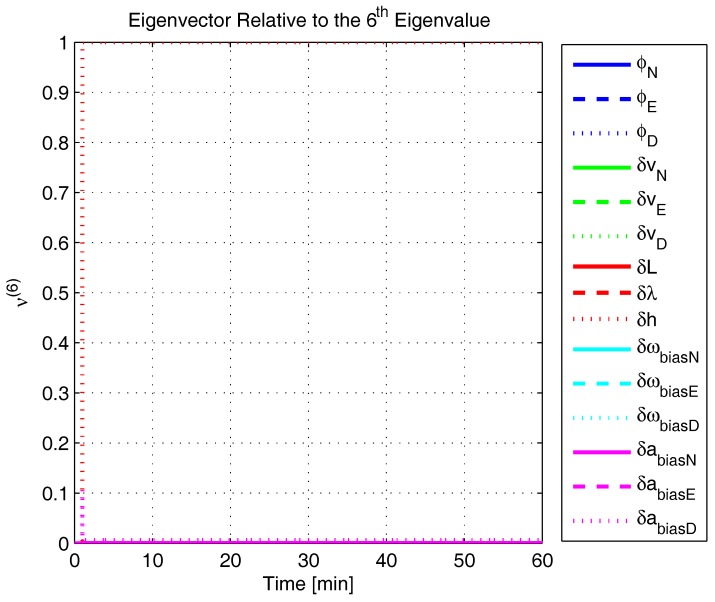
Eigenvector relative to the sixth eigenvalue in the simulated test.

In order to shed more light onto the estimability of the SSAC error states, we calculated the eigenvalues and eigenvectors of the normalized error state covariance matrix, as suggested in (45) and (46). The computed eigenvalues (in descending order) and their corresponding eigenvectors (represented as multidimensional vectors) are given in Figure 7, Figure 8, Figure 9, Figure 10, Figure 11, Figure 12 and Figure 13. For clarity, the stationary numeric values (*t* = 60 min) of the eigenvalues and eigenvectors are summarized in Table 1 and Table 2.

Looking at Figure 7, it is possible to notice that, in the steady state, six eigenvalues are conspicuously larger than the others. Recalling that large eigenvalues indicate states (or linear combination of states) that are poorly estimable, it is straightforward to infer that the latter correspond to the six unobservable modes (and consequently to the six unobservable subspaces) derived in Section 2. We can also observe that all six largest eigenvalues virtually possess the same magnitude and that their summation, as expected, approaches 15 (the order of the system).

If we look at the eigenvectors associated with the three largest eigenvalues of the covariance matrix, namely $\nu^{\left(1\right)}$ to $\nu^{\left(3\right)}$, we will notice that, in the steady state, they are directed in the halfway between $\varphi_{D}$ and $\delta \omega_{biasE}$, $\varphi_{N}$ and $\delta a_{biasE}$ and $\varphi_{E}$ and $\delta a_{biasN}$, respectively. This implies, in other words, that a portion of all of these error states can be effectively estimated by the filter, corroborating our preceding conclusions. By referring now to the eigenvectors of the next three largest eigenvalues $\nu^{\left(4\right)}$ to $\nu^{\left(6\right)}$, we notice that they point in the exact direction of the position errors. Once again, this is clear evidence that the position errors are the sole “non-estimable” error states in the SSAC problem.

A very interesting behavior that is also worth of remark is the “X”-shape pattern originated in Figure 9 and Figure 10. As can be noticed, from a given instant (approximately 50 min of the simulation), the eigenvectors $\nu^{\left(2\right)}$ and $\nu^{\left(3\right)}$ had their “estimability” directions interchanged. We have only been able to observe this pattern because, in this paper, the eigenvalues and eigenvectors of the covariance matrix were evaluated during the whole SSAC process. If we had evaluated the eigenvalues and eigenvectors at a specific time instant, as did Fang et al. [54,63], close for instance, to the X-shape pattern, we could be led to wrongly conclude that the estimability deficiency of $\varphi_{N}$ and $\delta a_{biasE}$ is related to that of $\varphi_{E}$ and $\delta a_{biasN}$. Actually, this is not the case. As we have seen in Section 2, the latter error states belong to separate and uncoupled unobservable subspaces. The fact that $\nu^{\left(2\right)}$ has its “estimability” direction modified over time should be understood together with the chances in $\nu^{\left(3\right)}$. Actually, if we vectorially sum $\nu^{\left(2\right)}$ and $\nu^{\left(3\right)}$, we will see that the obtained directions remain all constant over time, as the X-shape pattern had never occurred.

A reasonable explanation for the emergence of the aforementioned X-shape patterns in Figure 9 and Figure 10 is related to the mathematical description of the error state covariance matrix eigenvalues. As originally demonstrated by Ham and Brown [39], the (multiple) eigenvalues of the error state covariance matrix actually correspond to the variances of the (also multiple) SSAC error states (or linear combinations of SSAC error states). Due to the stochastic nature of the SSAC problem, however, these linear combinations are completely random, which means that their scalar components may well vary over time (the fact that these linear combinations may be randomly formed does not mean that the observability deficiency of the SSAC problem is indistinctly shared between states). See Equations (1), (2) and (9) of [39] for a more detailed mathematical description of the eigenvalues significance for stochastic problems.

Still regarding the obtained results, they also allow us to explain why the down angular rate sensor bias is often considered “non-estimable” in the literature [30,32,34,48,54]. As can be noticed in Figure 8, $\delta \omega_{biasD}$ possess, in fact, a relatively low estimability in the first 10 min of the simulation. The latter, however, does not mean that the down angular rate sensor bias is “non-estimable”. On the contrary, this actually reflects the already well-known $\delta \omega_{biasD}$ dependency on higher velocity error time derivatives (see (24) for details). Over the time, it is possible to see that the eigenvalue relative to $\delta \omega_{biasD}$ asymptotically approaches zero, which is the correct behavior, given that $\delta \omega_{biasD}$ is “estimable” in the problem.

Finally, it is worth mentioning that only the eigenvectors associated with the six largest eigenvalues of the covariance matrix have been presented in this section. The remaining nine eigenvectors (corresponding to the “observable” modes of the problem) have not been presented due to space constraints, but it is possible to prove that they point (in the form of linear combinations) in the directions of all SSAC error states, but the position errors, which, as already demonstrated, are the sole “non-estimable” states in the problem.

## 4. Experimental Results

**Figure 14:**
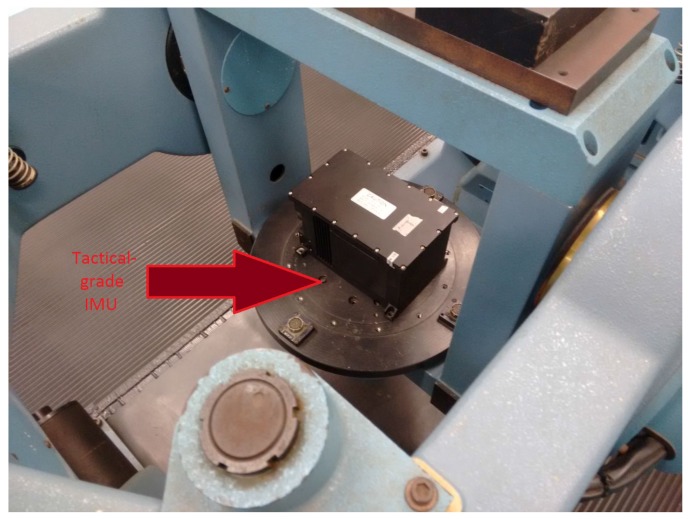
Tactical-grade IMU mounted on a three-axis rotary table.

Merely to validate the propriety of the observability/estimability analyses conducted so far, we repeated the simulated test, now with real datasets gathered from a tactical-grade inertial measurement unit (IMU). The employed IMU, specified in Table 3, was mounted aligned to the navigation frame, on a three-axis rotary table available at the “Identification, Navigation, Control and Simulation Laboratory” (LINCS) of the “Institute of Aeronautics and Space” (IAE), in São José dos Campos, Brazil, as shown in Figure 14. The inertial sensor outputs were processed at a cadence of 100 Hz (corresponding to the IMU sampling rate) and the Kalman filter at 1 Hz. For the purpose of the test, ${\hat{x}}_{0}$, $P_{0}$, $Q_{P}$ and $R_{M}$ were initialized as follows,

(47) $${\hat{x}}_{0}=0_{15\times 1}$$

(48) $$\begin{array}{c} P_{0}=\mathrm{diag}[{\left(0.1^{\circ }\right)}^{2}\;{\left(0.1^{\circ }\right)}^{2}\;{\left(5^{\circ }\right)}^{2}\;{\left(0.1\;m/s\right)}^{2}\;{\left(0.1\;m/s\right)}^{2}\;\\ {\left(0.1\;m/s\right)}^{2}\;{\left(0.0001^{\circ }\right)}^{2}\;{\left(0.0001^{\circ }\right)}^{2}\;{\left(10\;m\right)}^{2}\;{\left(0.5^{\circ }/h\right)}^{2}\;\\ {\left(0.5^{\circ }/h\right)}^{2}\;{\left(0.5^{\circ }/h\right)}^{2}\;{\left(0.5\;\mathrm{mg}\right)}^{2}\;{\left(0.5\;\mathrm{mg}\right)}^{2}\;{\left(0.5\;\mathrm{mg}\right)}^{2}] \end{array}$$

(49) $$\begin{array}{c} Q_{P}=\mathrm{diag}[{\left(0.01^{\circ }/\sqrt{h}\right)}^{2}\;{\left(0.01^{\circ }/\sqrt{h}\right)}^{2}\;{\left(0.01^{\circ }/\sqrt{h}\right)}^{2}\;\\ {\left(0.03\;\mathrm{mg}/\sqrt{\mathrm{Hz}}\right)}^{2}\;{\left(0.03\;\mathrm{mg}/\sqrt{\mathrm{Hz}}\right)}^{2}\;{\left(0.03\;\mathrm{mg}/\sqrt{\mathrm{Hz}}\right)}^{2}] \end{array}$$

(50) $$R_{M}=\mathrm{diag}\left[\begin{array}{ccc} {\left(0.1m/s\right)}^{2} & {\left(0.1m/s\right)}^{2} & {\left(0.1m/s\right)}^{2} \end{array}\right]$$

where the variances of $R_{M}$ in (50) were defined based on intuitive judgment.

**Figure 15:**
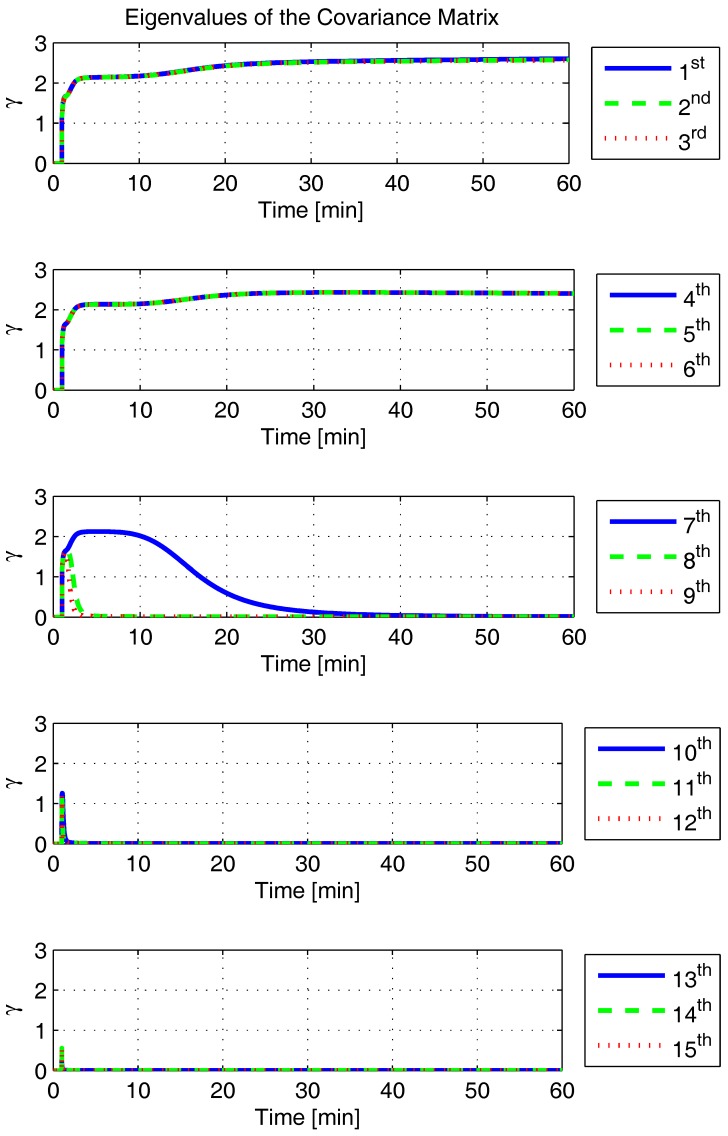
Eigenvalues of the covariance matrix in the experimental test.

**Figure 16:**
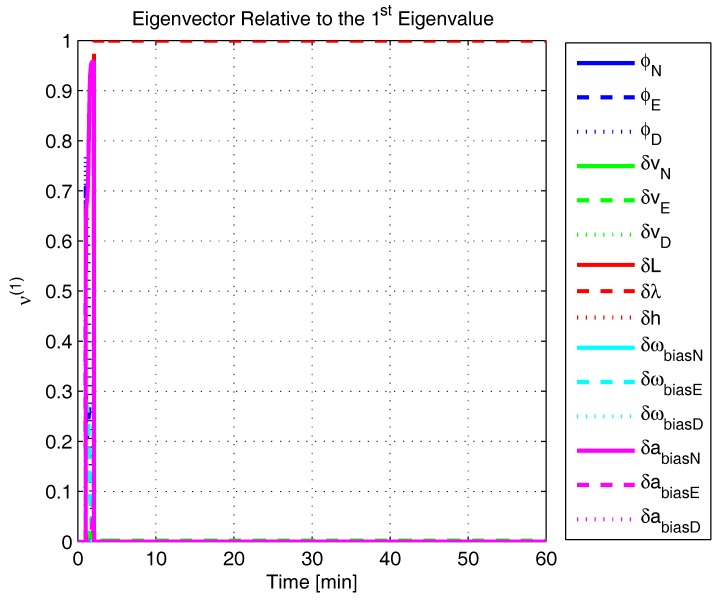
Eigenvector relative to the first eigenvalue in the experimental test.

**Figure 17:**
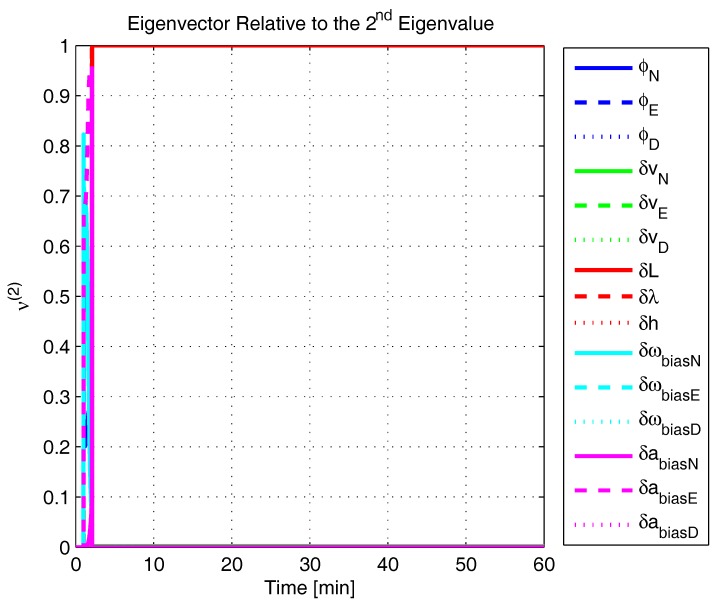
Eigenvector relative to the second eigenvalue in the experimental test.

**Figure 18:**
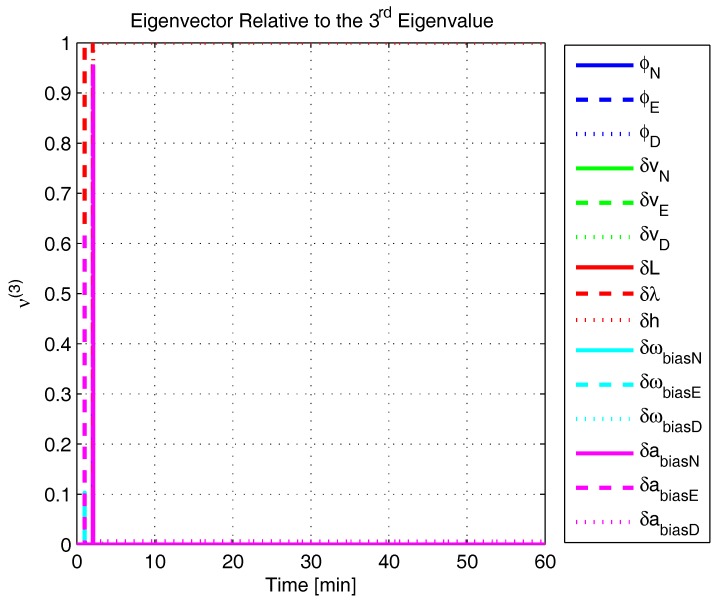
Eigenvector relative to the third eigenvalue in the experimental test.

**Figure 19:**
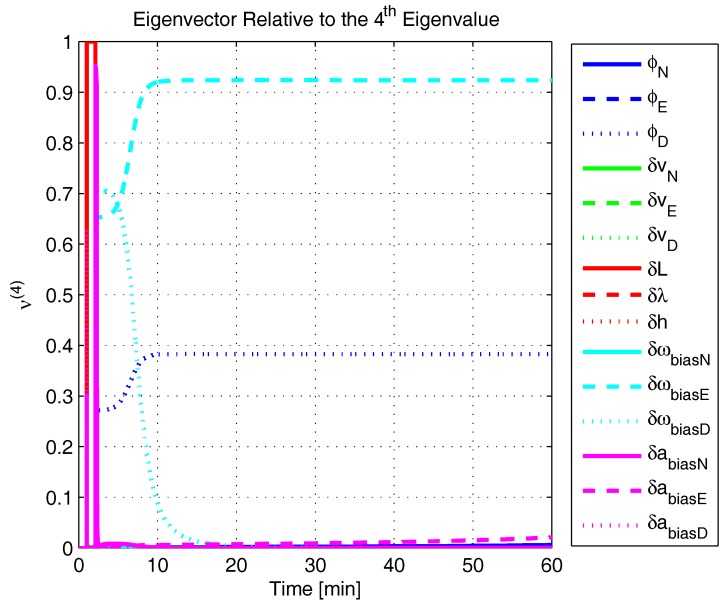
Eigenvector relative to the fourth eigenvalue in the experimental test.

**Figure 20:**
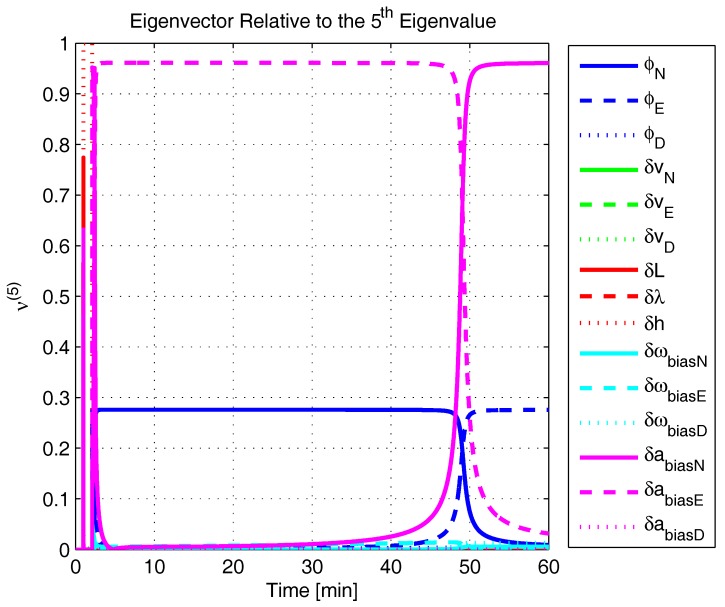
Eigenvector relative to the fifth eigenvalue in the experimental test.

**Figure 21:**
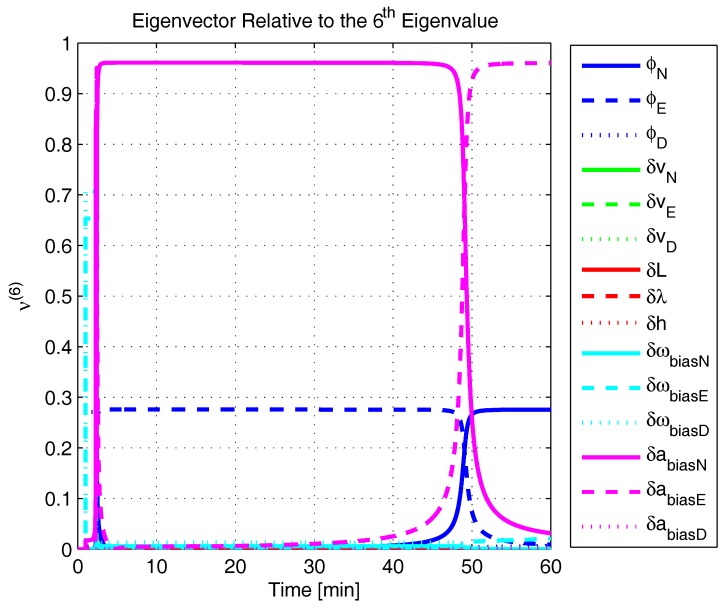
Eigenvector relative to the sixth eigenvalue in the experimental test.

The obtained eigenvalues (in descending order) and their corresponding eigenvectors are given in Figure 15, Figure 16, Figure 17, Figure 18, Figure 19, Figure 20 and Figure 21. For clarity, the stationary numeric values (*t* = 60 min) of the eigenvalues and eigenvectors are summarized in Table 4 and Table 5.

As can be seen, the behavior of the covariance matrix eigenvalues in the experimental test proved to be very similar to what has been observed in the simulated test, including the steady state values reached by the eigenvector directions. Concerning the latter, an important remark is required. The current literature does not provide us with much insight on the mechanisms that govern the steady state estimation accuracy of unobservable error states. What is known is that the final accuracy reached by their estimates is very dependent on the initial choice of the error state covariance matrix [3,14,41,46,48]. As suggested by Wu et al. [14], this is mainly due to the fact that the SSAC problem is a nonlinear estimation problem, which is generally attacked by linearization-based methods. Still, according to these authors, the observability characteristics of the original nonlinear system may be quite different from the corresponding linearized system, which means that when a Kalman filter is designed for the SSAC process, the estimator tends to converge to one of the unobservable states, depending on the estimator settings.

In order to observe the aforementioned behavior, we repeated the experimental test, by arbitrarily modifying the initial error state covariance matrix, as follows,

(51) $$\begin{array}{c} P_{0}=\mathrm{diag}[{\left(0.2^{\circ }\right)}^{2}\;{\left(0.2^{\circ }\right)}^{2}\;{\left(10^{\circ }\right)}^{2}\;{\left(0.15\;m/s\right)}^{2}\;{\left(0.15\;m/s\right)}^{2}\;\\ {\left(0.15\;m/s\right)}^{2}\;{\left(0.0001^{\circ }\right)}^{2}\;{\left(0.0001^{\circ }\right)}^{2}\;{\left(10\;m\right)}^{2}\;{\left(0.625^{\circ }/h\right)}^{2}\;\\ {\left(0.625^{\circ }/h\right)}^{2}\;{\left(0.625^{\circ }/h\right)}^{2}\;{\left(0.875\;\mathrm{mg}\right)}^{2}\;{\left(0.875\;\mathrm{mg}\right)}^{2}\;{\left(0.875\;\mathrm{mg}\right)}^{2}] \end{array}$$

**Figure 22:**
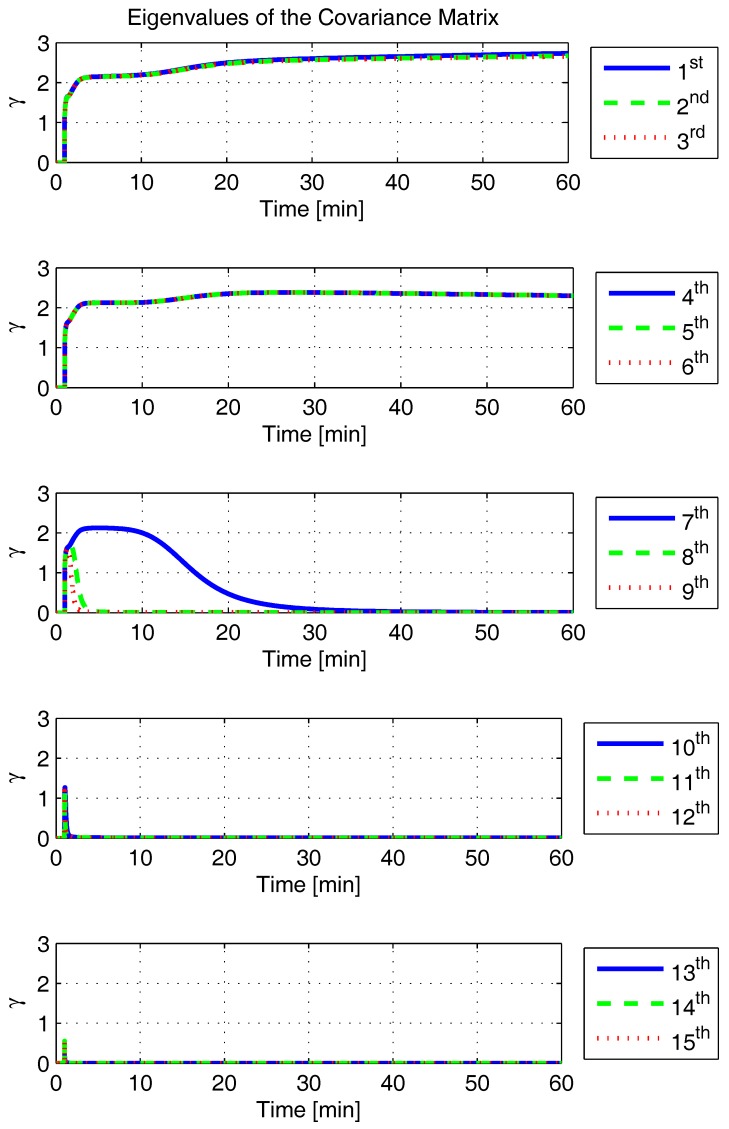
Eigenvalues of the covariance matrix in repeated experimental test.

**Figure 23:**
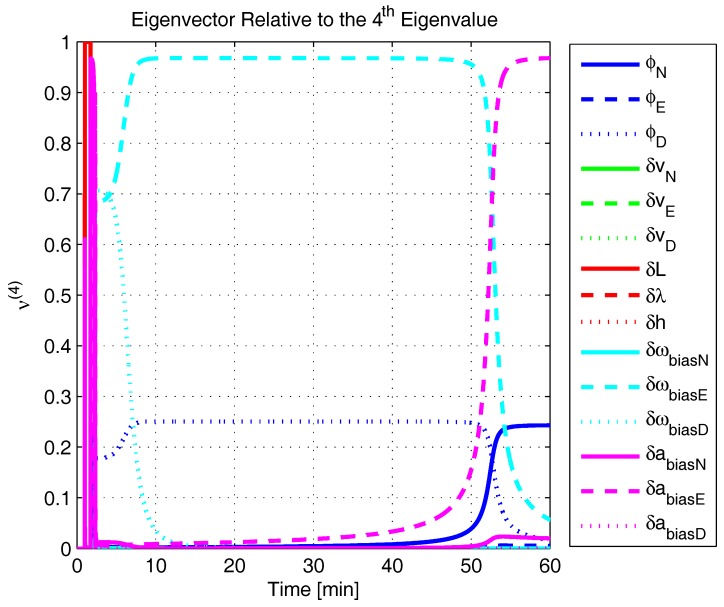
Eigenvector relative to the fourth eigenvalue in the repeated experimental test.

**Figure 24:**
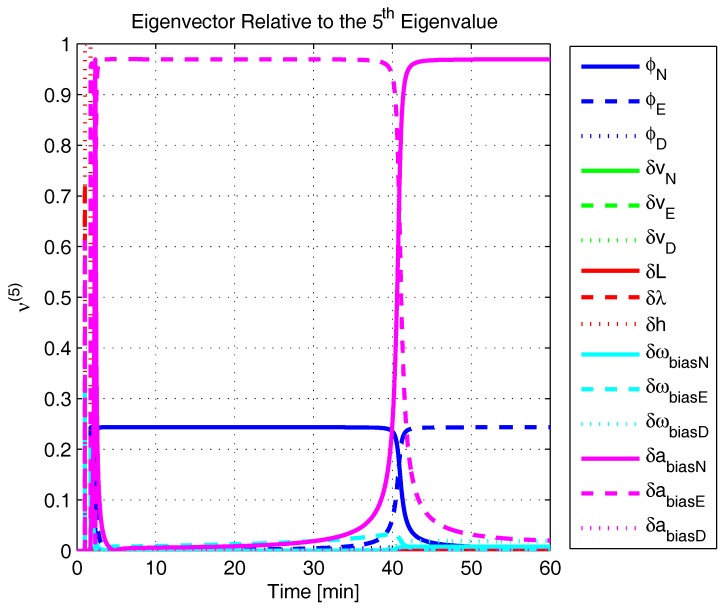
Eigenvector relative to the fifth eigenvalue in the repeated experimental test.

**Figure 25:**
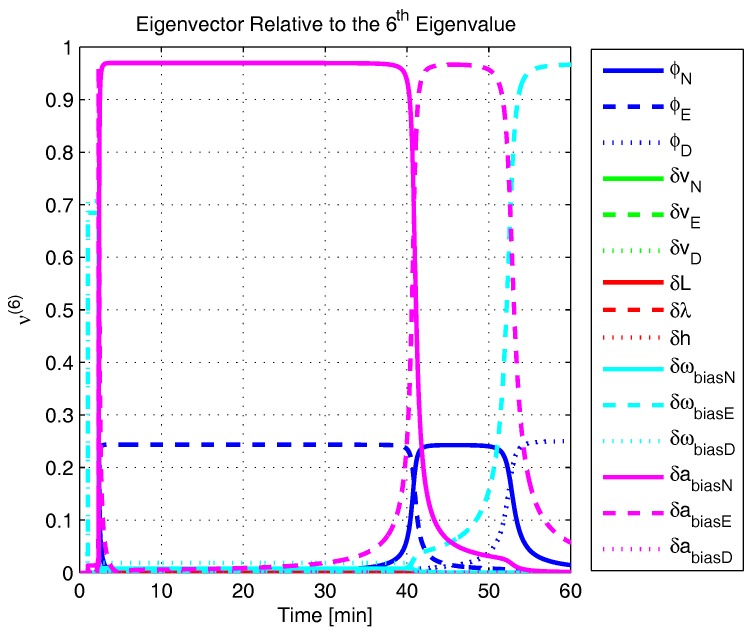
Eigenvector relative to the sixth eigenvalue in the repeated experimental test.

Figure 22, Figure 23, Figure 24 and Figure 25 and Table 6 and Table 7 depict the most representative results originated in the repeated experimental test (eigenvectors corresponding to the position errors have been omitted here, for brevity).

As can be verified, the initial choice of the error covariance matrix, in fact, is interfered on the steady state values reached by the eigenvector directions and, consequently, on the estimability of the error states. From Figure 23, Figure 24 and Figure 25, for instance, it is possible to notice that the inertial sensor bias estimates were hampered in comparison to what has been obtained in the original experimental test (observe that the eigenvector components in the direction of the inertial sensor biases approached unity and, hence, approached complete “non-estimability”). Obviously, the lower the estimability of the inertial sensor biases, the worse the estimation scenario for the alignment errors (recall that the latter are uniquely constrained by the former). See [36] for details.

Finally, it is worth mentioning that all considerations made so far are independent of the orientation that the SINS is simulated or tested. This can be justified by recalling that the estimation algorithms that gave rise to the observability/estimability analysis performed in this paper were obtained by resolving the error state variables in the navigation frame (*l*-frame). Thus, when an SINS is simulated or tested at an arbitrary orientation, it is obvious that eventual biases in all inertial sensor axes will experience a small portion of “non-estimability”. However, it is possible to prove that the vectorial summation of the latter will point exactly in the east direction (for the angular rate sensor biases) and in the north and east directions (for the accelerometer biases), exactly as predicted in (34)–(36). The readers are invited to carefully analyze the works of Rothman et al. [62] and Jiang and Lin [31], where this issue is systematically addressed.

## 5. Conclusions

In this paper, the second part of a study aiming at the error state selection for SSAC Kalman filters has been presented. Complementary to [36], wherein the issue has been investigated through the derivation of estimation algorithms, here we focused on the observability/estimability properties of the system.

Based on the SSAC dynamic and measurement models, we firstly handled the SSAC observability problem by deterministic means, establishing the number of unobservable modes in the latter. By analytically manipulating the estimation algorithms derived in [36], we introduced some new findings, demonstrating that the SSAC unobservable modes are, in fact, linear combinations of the alignment errors, inertial sensor biases, and position errors, which can be grouped into different and uncoupled subspaces. As a straightforward consequence of the analysis, all SSAC error states (except the velocity errors) were proven to be individually unobservable in the problem.

On the basis of the performed verifications, the issue of reducing the SSAC Kalman filter dimensionality was addressed from the observability standpoint, and several drawbacks of the approach were evidenced. In this sense, basic prerogatives of very recognizable works in the area were shown to be inadequate. In order to solve the problem, the concept of “estimability” was recalled from the literature, proving to be a much more suitable parameter for filter-order reduction.

In an attempt to ascertain the estimability of the SSAC problem, we firstly resorted to the well-known covariance analysis, demonstrating that, except for the position errors, all remaining SSAC error states can be minimally estimated by the filter. Based on Baram and Kailath’s statement that reduced-order filters are to be implemented when the system is non-completely estimable (and not, non-completely observable), we concluded that the position errors are the sole states liable to be adequately dropped from the SSAC Kalman filter. The preceding verifications corroborated the conclusions made in [36], where a 12-state Kalman filter was considered the optimal error state selection for SSAC purposes.

As an additional contribution of this paper, the investigation of the individual estimability of the SSAC error states has also been conducted. For the purpose of the latter, the convergence rate and steady state values of the major diagonal terms of the covariance matrix have been examined. Additionally, the eigenvalues and eigenvectors of the normalized covariance matrix were also evaluated, providing us with much more insight into the estimability of linear combination of states. As we verified, the estimability of the SSAC error states is closely connected to the choice of the Kalman filter settings, especially the initial error state covariance matrix, which figures as an important research topic for future investigation.

An additional comment worthy of noting concerns the practical consequences of having states in the Kalman filter that are not completely estimable (precisely the case of the SSAC problem). One consequence is that it is not straightforward to determine how “well estimated” these states can be. Additionally, it is very probable that, if a state is not completely estimable (or otherwise, if it is estimable, but has been inadvertently removed from the filter) and it is dynamically coupled to some state variable, the filter will produce an unstable (or biased) estimate for this variable. Figures 1–3 of the first part of this study [36] confirm the aforementioned behavior.

Finally, it is important to emphasize that all verifications and conclusions outlined so far are restricted to the alignment and calibration process of stationary autonomous (non-aided) SINS. As mentioned in Section 1, this stationary self-alignment and calibration (SSAC) process is confined to very particular cases (satellite launch vehicles and ballistic missiles), whose strategic nature prevents the use of external aiding devices and more modern alignment techniques (“in-flight”, transfer and multiposition alignment). For situations, however, wherein it is possible to perform the alignment under non-stationary conditions, the full-order filter (with 15-states) becomes a more interesting alternative than the 12-state Kalman filter supported in this paper. As analyzed by [20,45,46,68,76,77,78,79], this is mainly due to the application of induced vehicle manoeuvres and to the use of additional sensor measurements, which excite the latent modes of the system, improving the estimability of all error states (especially the position errors, which are, as we saw, non-estimable under stationary conditions). In this sense, it is found that, the greater the maneuverability of the vehicle, or the amount of external aiding sensors (multiple GNSS antennas, for instance), the better the estimator’s performance [80,81].

As suggestions for future works, we intend to examine the mechanisms that govern the steady state estimation accuracy of the SSAC unobservable modes. In parallel, the use of different measurement selections for the SSAC problem seems to be a subject definitely worthy of investigation. Finally, another front of promising studies concerns the possibility of obtaining reduced-order Kalman filters for the SSAC problem by reducing the associated Riccati equation instead of reducing the dynamic error model. Similar ideas have been successfully employed in [37,82].

## Figures and Tables

**Table 1:** Stationary eigenvalues (*t* = 60 min) of the covariance matrix in the simulated test.

Eigenvalue Order	Numeric Value
1st	2.4985
2nd	2.4985
3rd	2.4985
4th	2.4985
5th	2.4985
6th	2.4985
7th	0.0087
8th	0.0000
9th	0.0000
10th	0.0000
11th	0.0000
12th	0.0000
13th	0.0000
14th	0.0000
15th	0.0000

**Table 2:** Stationary eigenvectors (*t* = 60 min) relative to the largest eigenvalues in the simulated test.

Eigenvector Direction	Numeric Value					
	$\nu^{\left(1\right)}$	$\nu^{\left(2\right)}$	$\nu^{\left(3\right)}$	$\nu^{\left(4\right)}$	$\nu^{\left(5\right)}$	$\nu^{\left(6\right)}$
$\varphi_{N}$	0.0046	0.0108	0.2757	0.0000	0.0000	0.0000
$\varphi_{E}$	0.0001	0.2757	0.0108	0.0000	0.0000	0.0000
$\varphi_{D}$	0.3829	0.0000	0.0045	0.0000	0.0000	0.0000
$\delta v_{N}$	0.0000	0.0000	0.0000	0.0000	0.0000	0.0000
$\delta v_{E}$	0.0000	0.0000	0.0000	0.0000	0.0000	0.0000
$\delta v_{D}$	0.0000	0.0000	0.0000	0.0000	0.0000	0.0000
δL	0.0000	0.0000	0.0000	0.0000	1.0000	0.0000
δλ	0.0000	0.0000	0.0000	1.0000	0.0000	0.0000
δh	0.0000	0.0000	0.0000	0.0000	0.0000	1.0000
$\delta \omega_{biasN}$	0.0000	0.0057	0.0002	0.0000	0.0000	0.0000
$\delta \omega_{biasE}$	0.9236	0.0002	0.0165	0.0000	0.0000	0.0000
$\delta \omega_{biasD}$	0.0001	0.0133	0.0005	0.0000	0.0000	0.0000
$\delta a_{biasN}$	0.0005	0.9603	0.0376	0.0000	0.0000	0.0000
$\delta a_{biasE}$	0.0163	0.0376	0.9603	0.0000	0.0000	0.0000
$\delta a_{biasD}$	0.0000	0.0000	0.0000	0.0000	0.0000	0.0063

**Table 3:** Tactical-grade IMU technical specifications.

Parameters	Accelerometer Channel	Angular Rate Sensor Channel
Cadence of acquisition	100 Hz	100 Hz
Random walk	0.03 mg/$\sqrt{\mathrm{Hz}}$	0.01 ${}^{\circ }$/$\sqrt{h}$
Scale factor	100 ppm	70 ppm
Bias - short term	0.1 mg	0.1 ${}^{\circ }$/h
Bias - long term	0.2 mg	(not specified)
Dynamic range	40 g	600 ${}^{\circ }$/s

**Table 4:** Stationary eigenvalues (*t* = 60 min) of the covariance matrix in the experimental test.

Eigenvalue Order	Numeric Value
1st	2.6035
2nd	2.5742
3rd	2.5693
4th	2.4024
5th	2.4024
6th	2.4024
7th	0.0141
8th	0.0127
9th	0.0107
10th	0.0082
11th	0.0000
12th	0.0000
13th	0.0000
14th	0.0000
15th	0.0000

**Table 5:** Stationary eigenvectors (*t* = 60 min) relative to the largest eigenvalues in the experimental test.

Eigenvector Direction	Numeric Value					
	$\nu^{\left(1\right)}$	$\nu^{\left(2\right)}$	$\nu^{\left(3\right)}$	$\nu^{\left(4\right)}$	$\nu^{\left(5\right)}$	$\nu^{\left(6\right)}$
$\varphi_{N}$	0.0000	0.0000	0.0000	0.0059	0.0091	0.2757
$\varphi_{E}$	0.0000	0.0000	0.0000	0.0002	0.2758	0.0090
$\varphi_{D}$	0.0000	0.0000	0.0000	0.3829	0.0001	0.0063
$\delta v_{N}$	0.0000	0.0019	0.0000	0.0000	0.0000	0.0000
$\delta v_{E}$	0.0000	0.0000	0.0000	0.0000	0.0000	0.0000
$\delta v_{D}$	0.0020	0.0000	0.0023	0.0000	0.0000	0.0000
δL	0.0000	1.0000	0.0000	0.0000	0.0000	0.0000
δλ	0.0000	0.0000	0.0000	0.0000	0.0000	0.0000
δh	1.0000	0.0000	1.0000	0.0000	0.0000	0.0000
$\delta \omega_{biasN}$	0.0000	0.0000	0.0000	0.0000	0.0057	0.0002
$\delta \omega_{biasE}$	0.0000	0.0000	0.0000	0.9236	0.0001	0.0210
$\delta \omega_{biasD}$	0.0000	0.0000	0.0000	0.0002	0.0133	0.0004
$\delta a_{biasN}$	0.0000	0.0000	0.0000	0.0006	0.9606	0.0315
$\delta a_{biasE}$	0.0000	0.0000	0.0000	0.0210	0.0315	0.9604
$\delta a_{biasD}$	0.0000	0.0000	0.0061	0.0000	0.0000	0.0000

**Table 6:** Stationary eigenvalues (*t* = 60 min) of the covariance matrix in the repeated experimental test.

Eigenvalue Order	Numeric Value
1st	2.7357
2nd	2.6725
3rd	2.6549
4th	2.3021
5th	2.3021
6th	2.3021
7th	0.0099
8th	0.0088
9th	0.0069
10th	0.0051
11th	0.0000
12th	0.0000
13th	0.0000
14th	0.0000
15th	0.0000

**Table 7:** Stationary eigenvectors (*t* = 60 min) relative to the largest eigenvalues in the repeated experimental test.

Eigenvector Direction	Numeric Value		
	$\nu^{\left(4\right)}$	$\nu^{\left(5\right)}$	$\nu^{\left(6\right)}$
$\varphi_{N}$	0.2431	0.0050	0.0145
$\varphi_{E}$	0.0050	0.2435	0.0004
$\varphi_{D}$	0.0168	0.0001	0.2502
$\delta v_{N}$	0.0000	0.0000	0.0000
$\delta v_{E}$	0.0000	0.0000	0.0000
$\delta v_{D}$	0.0000	0.0000	0.0000
δL	0.0000	0.0000	0.0000
δλ	0.0000	0.0000	0.0000
δh	0.0000	0.0000	0.0000
$\delta \omega_{biasN}$	0.0002	0.0081	0.0000
$\delta \omega_{biasE}$	0.0568	0.0004	0.9664
$\delta \omega_{biasD}$	0.0004	0.0188	0.0000
$\delta a_{biasN}$	0.0200	0.9695	0.0016
$\delta a_{biasE}$	0.9680	0.0200	0.0573
$\delta a_{biasD}$	0.0000	0.0000	0.0000
